# Effects of a price cut reform on the cost and utilization of antidiabetic drugs in Korea: a national health insurance database study

**DOI:** 10.1186/s12913-018-3255-y

**Published:** 2018-06-08

**Authors:** Hae Sun Suh, Jee-Ae Kim, Iyn-Hyang Lee

**Affiliations:** 1 College of Pharmacy, Pusan National University, Busan, South Korea; 2 Health Insurance Review & Assessment Service, Wonju, South Korea; 3 College of Pharmacy, Yeungnam University, 280 Daehak-ro, Gyeongsan, Gyeongbuk-do 38541 South Korea

**Keywords:** Direct price control, Price cut, Pharmaceutical costs, Pharmaceutical utilization, Antidiabetics

## Abstract

**Background:**

Despite the potential widespread application and a significant need, the policy effectiveness of prescribed medications price controls has not been studied extensively. We aimed to explore the effects of a price cut introduced in April 1st of 2012 on the cost and utilization of antidiabetics in South Korea.

**Methods:**

We identified approximately four million outpatients who filed at least one diabetes-related claim during the index period (January 2010 to December 2012) using the National Health Insurance claims data. We performed interrupted time series analyses for cost and utilization of “overall,” “reduced price,” and “constant price” antidiabetics between January 2009 and June 2013, and measured the growth rate for incidents of medical and surgical procedures for diabetes-induced complications.

**Results:**

The segmented regression suggests that spending on overall and reduced price antidiabetics would drop by 6 and 23%, respectively; spending on constant price antidiabetics would rise by 16% in a year after the new pricing compared to if the policy were not in existence. There were a few immediate changes in utilization, and its trend indicated a significant decrease in reduced price antidiabetics and an increase in constant price antidiabetics. Incidents of medical and surgical procedures relating to diabetic complications were unaffected.

**Conclusions:**

The Korean price cut program contained costs by immediately reducing the cost of pharmaceuticals without any major signals associated with compromised clinical conditions in diabetic patients.

## Background

In many countries, healthcare authorities face challenges in containing expenditure on pharmaceuticals [1–4]. Governments are exploring more cost-effective strategies in regulating pharmaceuticals in a world of finite resources [5]. On the other hand, the “growing need for evidence-based healthcare” is leading to an increased demand for evidence that demonstrates the value of policies to governments [6–8]. In order to meet such societal goals, drug policies continue to evolve and are becoming increasingly complex [2, 9–13].

Along with cost-sharing schedules, price control policies are one of the most conventional strategies in drug policies [12, 14]. There is abundant evidence available for cost-sharing policies compared to price control strategies. Cost-sharing schemes have reduced drug expenditure by controlling public demand for pharmaceuticals [10, 15, 16]. However, obviously, excessive suppression of pharmaceutical use results in unwanted consequences in vulnerable populations (e.g., elderly, low income households). Reported consequences include an increase in institutionalization [17], emergency room visits [18], and physician visits [19], which imply the exacerbation of patient conditions and a decline in using essential medications [20], leading patients to suffer from more serious conditions.

Contrary to cost-sharing schemes, price control strategies work on the supply side of the pharmaceutical market, and as such, the pharmaceutical industry is the main stakeholder affected [13, 21]. Under price control strategies, governments set price limits, profit limits, and mark-up limits to restrain the industry from exploiting their monopolistic position in pricing [14]. Authorities in countries such as the United Kingdom, United States, France, and Italy negotiate pharmaceutical prices with the industry through strategies like price-volume agreements or risk-sharing schemes [22, 23]. At a national level, the United States is a unique market that allows the industry to set their price based primarily on the principle of market competition.

Guillen and Cabiedes [1] argued that the industry seemed to be extremely successful in seeking *“escape valves”* by selling more products and/or selling premium products. Thus, it is crucial to study how pricing policies work [24]. Despite the potential widespread application and a significant need, the policy effectiveness of price controls has not been studied extensively. Pertinent policy studies are surprisingly limited and existing evidence is mainly around how reference pricing works [15, 25, 26]. Lee et al. [15] systematically reviewed studies on pharmaceutical policies over the past 30 years and found 25 studies that examined price controls using robust scientific methods. Of those, sixteen studies explored reference pricing programs and only nine studies explored other types of pricing programs. Of the nine studies, a single study from Ireland reported significant savings in expenditure after a reduction in the wholesale margin. Cochrane’s updated review only found two policy studies on maximum prices or index pricing and concluded uncertainty in the effect of pricing policies “due to sparse evidence” [26].

The purpose of this study is to explore the impact of a direct price cut on pharmaceutical costs, utilization, and any consequences that possibly compromised the public health in South Korea, where a price cut schedule was implemented in April 2012. With this new policy, the Korean government aimed to contain pharmaceutical costs with few changes in the patients’ utilization of pharmaceuticals, and thus, without compromising public health. The price cut was also expected to improve health disparities by lowering the price of medications which became more affordable to those at the margins of society with limited ability to access medications. Our study focuses on antidiabetics, which is a medication used for diabetes, a chronic disease, and examines the impacts of the policy for over one year after policy implementation.

## Methods

### Policy intervention and study design

Since 2006, Korea has had a drug pricing system in which the prices for original pharmaceuticals declines to 80% of the on-patent prices when the patent expires. Prices for generics were set to 90% of off-patent prices and varied depending on when they entered the market; the earlier they entered, the higher the price. In April 1st of 2012, the government introduced a new pricing system, known as the “Single Price System (SPS).” Prices for off-patents were reduced from 80 to 70% of the on-patent prices, and generics were uniformly priced at 85% of their off-patent counterparts (equivalent to 59.5% of the on-patent price). One year after the expiration of patents, all pharmaceuticals including off-patents and their generics were priced at 53.55% of the on-patent prices [27]. We used an interrupted time series design to test the impact of the SPS on the cost and utilization of antidiabetics. We built a time series for each of the outcome variables over 54 months (4.5 years) between January 2009 and June 2013. The intervention policy occurred at the 40th month, and there were 15 months in the post-intervention period.

### Data source and population

We examined the administrative National Health Insurance (NHI) claims databases of the Health Insurance Review & Assessment Service (HIRA) to identify the study population. The Korean healthcare system is composed of a mandatory social insurance plan, the NHI, and a medical aid program (Medical Aid, MedAid) that provides additional benefits to low income households. Since 2000, the two national health plans have covered the entire population—about 97% by the NHI and 3% by MedAid [28]. Since Korea has a mandatory health security system for national health insurance, the NHI claims that the database contains all medical and prescription drug claims records for the entire population in Korea.

Subjects for this study are adult beneficiaries (≥20 years old) covered by either NHI or MedAid who had at least one claim with the diagnosis of diabetes mellitus in an outpatient setting during the reference period between January 1, 2010 and December 31, 2012. The subject included in the study were cases with Type 2 Diabetes Mellitus as diagnosis using the codes of E11 (non-insulin-dependent diabetes mellitus) or E14 (unspecified diabetes mellitus excluding insuline-dependent diabetes mellitus, etc.) in the 6th Korean Standard Classification of Diseases and Causes of Death, an official Korean version of the 10th version of the International Classification of Diseases (ICD-10) [29]. After specifying the study population, we established a dataset composed of the study subjects’ medical and drug claims between January 1, 2009 and June 31, 2013.

### Identification and classification of antidiabetics

We defined antidiabetics as medications in the WHO Anatomic Therapeutic Chemical (ATC) group A10. To identify A10 medications from the claims, we used the Korean National Drug Classification system and searched for the drug group “396” which corresponds to the ATC group A10 [30]. We identified 104 insurance codes of active ingredients for antidiabetics from the Korean drug benefits list and finally included 97 codes by eliminating seven which were deleted before January 2008 from the benefit list. Of those, we found 32 ingredients that had their prices cut by the SPS in April 2012 and grouped these as “antidiabetics with reduced price (antidiabetics_p-cut_).” The rest of the antidiabetics were grouped as “antidiabetics with constant price (antidiabetics_p-keep_).”

### Outcome measures

*Primary outcomes* were the monthly cost and utilization of antidiabetics after the price cut. *Secondary outcomes* were individual cost, utilization of antidiabetics, and incidents of medical and surgical procedures for diabetic complications in the study sample. Each measure was operationally defined as in Table 1.

**Table 1:** Definition of outcome measures

Outcomes	Measures	Definition
Primary outcomes	Pharmaceutical cost^a^ (total)	• Monthly cost of antidiabetics
	Pharmaceutical utilization (total)	• Monthly DDDs of antidiabetics
		• Monthly number of patients with at least one antidiabetics
Secondary outcomes	Pharmaceutical cost^a^ (per patient)	• Monthly cost per patient with antidiabetics
	Pharmaceutical utilization (per patient)	• Monthly DDDs per patient with antidiabetics
	Incidents of medical and surgical procedures relating to diabetic complications	• *Diabetic retinopathy* including 3 procedures (panretinal photocoagulation, and vitrectomy)
		• *Diabetic cataract* including 4 procedures (extracapsular or intracapsular extraction, pars plana lensectomy, phacoemulsification, and surgery after cataract)
		• *Diabetic nephropathy* including 20 procedures (e.g., AV shunt, fistula formation or various intravenous catheter insertions for hemodialysis, kidney transplant)
		• *Cardiovascular complications of diabetics* including 15 procedures (e.g., percutaneous coronary intervention, coronary artery bypass grafting)
		• *Diabetic foot lesions* including 31 procedures (e.g., limb amputation, atherectomy)

^a^Cost in Korean won, KRW (1 US dollar ≒ 1000 KRW); DDD = defined daily dose (In the case of pharmaceutical items without DDD information, for example, combinations of oral blood glucose lowering drugs (A10BD), we divided the total quantity consumed by the standard daily dosage designated for adults in the Korean official labels to compute total DDDs for that item)

### Statistical analysis

We present descriptive statistics for all variables. We measured annual growth rates in incidents of medical and surgical procedures relating to diabetic complications, including diabetic retinopathy, diabetic cataract, diabetic nephropathy, cardiovascular complications, and diabetic foot lesions. We examined the time-series data of interests graphically and established segmented regression models to assess statistical significance of the policy effects. We measured policy effects as a change in the slope and level of the time series [31]. In the time series analysis, any change in the slope indicates a long term effect of the policy, and any change in the level stands for an abrupt effect of the policy. We used the Durbin–Watson test to assess serial correlation and estimated the regression coefficients with either an ordinary least squares (OLS) or a first order autocorrelation maximum likelihood estimate (AR) depending on the significance of serial correlations [32]. Using the SAS autoregression procedure, outcome variables were analyzed by time series methods. The final model for each time series was selected based on the minimum Akaike Information Criterion score [33]. We carried out residual analyses based on autocorrelation plots and partial autocorrelation plots. Assessed models were chosen from those that resulted in residuals that were not significantly different from white noise.

Through repeated model specifications, we were able to build the final model for the time series of interest as below.

$$ {\mathrm{Y}}_t={\beta}_0+{\beta_1}^{\ast }{\mathrm{time}}_t+{\beta_2}^{\ast }{\mathrm{OPIP}}_t+{\beta_3}^{\ast}\mathrm{time}\ {\mathrm{after}\ \mathrm{OPIP}}_t+{\beta_4}^{\ast }{\mathrm{SPS}}_t+{\beta_5}^{\ast}\mathrm{time}\ \mathrm{after}\ {\mathrm{SPS}}_t+{\beta_6}^{\ast}\mathrm{Feb}+{\upvarepsilon}_t $$

Where ***Y***_***t***_ is the outcome variable (as defined in “Outcome measures”) in month *t*; ***time*** is a continuous variable indicating time in months from January 2009 to June 2013; ***OPIP*** is a dummy variable for time *t* occurring before (policy = 0) or after (policy =1) the launch of the Outpatient Prescription Incentive Program (OPIP, October 2010); ***time after OPIP*** is a continuous variable coded 0 before the launch of the OPIP, and then counted 1 in October 2010 to 33 in June 2013; ***SPS*** is a dummy variable for time *t* occurring before (price cut = 0) or after (price cut =1) the launch of the SPS scheme (April 2012); ***time after SPS*** is a continuous variable coded 0 before the scheme, and then counted 1 in April 2012 to 15 in June 2013; and ***Feb*** is a dummy variable indicating the month of February in each year (February = 1, other months = 0). In the model, ***β***_***0***_ estimates the baseline levels of the outcome variables; ***β***_***1***_ estimates the changes in the outcome variables before the OPIP, i.e. the baseline trends; ***β***_***2***_ estimates the level changes in the outcome variables after the OPIP; ***β***_***3***_ estimates the changes in the trend of the outcome variables after the OPIP; ***β***_***4***_ estimates the level changes in the outcome variables after the SPS; ***β***_***5***_ estimates the changes in the trend of the outcome variables after the SPS and ***β***_***6***_ is a coefficient for the February variable.

The OPIP variables represent the Outpatient Prescription Incentive Program (OPIP), a policy introduced in October 2010 during the study period. The OPIP is an incentive program for prescribers who have achieved savings in their pharmaceutical expenditure compared to the year before [34]. This variable was introduced for covariate control since there is a possibility of its influence on pharmaceutical costs through changing prescribing behavior. The February variable is a dummy variable indicating whether the data is from February of each year. February has less days than other months so pharmaceutical utilization was observed to be low, and thus there was a need to control its influence on the estimation. We performed the analyses in SAS 9.4 (SAS Institute Inc., Cary, NC, USA). We determined statistical significance at *p*≤0.05.

## Results

### Study population demographics and descriptive summary of the data

Table 2 gives an overview of the study population. Approximately four million beneficiaries were identified as being diagnosed with diabetes mellitus, and had at least one claim during the index period. The study cohort was comprised of 53–54% women and 92% National Health Insurance beneficiaries (8% Medical Aid). The mean age of the cohort changed from 60 in 2009 to 62 in 2012. Total healthcare cost increased from 682 billion KRW in 2009 to 952 billion KRW in 2012. Total patient copayments grew from 51 billion KRW in 2009 to 55 billion KRW in 2010, and dropped by 10% to 50.5 billion KRW in 2012 after the introduction of the SPS scheme. While the quantity of antidiabetics prescribed increased from 84 to 97 million DDDs (Defined Daily Doses) during the study period, the cost spent on antidiabetics took a downturn from 37 to 36 billion KRW between 2011 and 2012 with the introduction of the SPS scheme.

**Table 2:** Population demographics and descriptive summary of data

	2009 April 2009 to March 2010	2010 April 2010 to March 2011	2011 April 2011 to March 2012	2012 April 2012 to March 2013
Beneficiaries, monthly mean ± SD	3,878,295 ± 80,558	4,013,070 ± 75,172	4,074,566 ± 47,546	4,050,415 ± 52,953
Men, %	46.1	46.6	46.9	47.1
Women, %	53.9	53.4	53.1	52.9
Age, mean ± SD	60.11 ± 12.93	60.72 ± 12.98	61.30 ± 12.95	61.95 ± 12.86
Medical Aid beneficiaries, %	8.03	8.12	7.96	7.73
Total healthcare costs, monthly mean ± SD (billion KRW)	681.80 ± 74.15	844.85 ± 53.19	924.49 ± 47.72	952.44 ± 54.81
Total copayments, monthly mean ± SD (billion KRW)	51.13 ± 2.71	55.07 ± 2.93	54.49 ± 2.43	50.50 ± 2.13
Total drug costs, outpatients, monthly mean ± SD (billion KRW)	290.28 ± 15.16	312.74 ± 15.97	327.62 ± 7.56	292.91 ± 8.55
Costs of antidiabetics, outpatients, monthly mean ± SD (billion KRW)	32.77 ± 1.78	35.30 ± 1.68	36.89 ± 0.86	35.75 ± 1.55
Costs in price reduced group	24.21 ± 1.01	25.28 ± 1.25	24.82 ± 0.97	18.44 ± 0.79
Costs in price constant group	8.56 ± 1.02	10.02 ± 0.57	12.07 ± 1.24	17.31 ± 1.85
DDDs of antidiabetics, monthly mean ± SD (million)	83.56 ± 4.22	88.53 ± 4.34	93.39 ± 2.15	97.12 ± 2.64
DDDs in price reduced group	71.75 ± 2.94	74.16 ± 3.56	75.39 ± 1.42	72.38 ± 2.25
DDDs in price constant group	11.81 ± 1.70	14.37 ± 0.86	18.00 ± 1.70	24.74 ± 2.27
Number of prescriptions with antidiabetics, monthly mean ± SD	1,520,685 ± 58,707	1,632,339 ± 57,761	1,762,863 ± 56,913	1,935,612 ± 58,314
Number of prescriptions in price reduced group	1,174,390 ± 34,270	1,226,601 ± 41,480	1,286,483 ± 22,929	1,316,360 ± 25,313
Number of prescriptions in price constant group	346,295 ± 27,689	405,739 ± 17,125	476,380 ± 36,017	619,221 ± 48,936

*SD* standard deviation, *KRW* Korean won (1000 KRW ≒ 1 US$ in January 2015), *DDD* defined daily dose

### Effects on the cost of antidiabetics

During the baseline period until the SPS, monthly average spending on antidiabetics_p-cut_ was stable at 24–25 billion KRW, but dropped by 28% to 18 billion KRW after the scheme (Table 2). In the regression model, a 4.8 billion KRW drop in level (*p* < 0.001) and a 0.02 billion KRW insignificant drop in slope were estimated (Table 3).

**Table 3:** Segmented regression coefficients for antidiabetics in outpatients by the price cut

Outcome variable		Policy group	Model	Coefficient								DW
				Baseline		Outpatient Prescription Incentive Program (OPIP)		Price cut (SPS)		February	AR(1)	
				*ß*_*0*_	*ß*_*1*_	*ß*_*2*_	*ß*_*3*_	*ß*_*4*_	*ß*_*5*_	*ß*_*6*_		
Costs (billion KRW)		Overall	AR	29.982^§^	0.295^§^	−0.984^#^	− 0.136^†^	− 4.384^§^	0.192^†^	−2.643^§^	0.282^#^	1.98
		Reduced	AR	23.738^§^	0.065^†^	1.357^§^	−0.185^§^	−4.809^§^	− 0.023	− 1.858^§^	0.277^#^	2.06
		Constant	AR	6.240^§^	0.229^§^	−2.264^§^	0.049	0.384	0.211^§^	−0.856^§^	− 0.080	1.80
Utiliz-ation	DDDs (million)	Overall	AR	79.447^§^	0.463^§^	−0.556	− 0.023	−1.058	− 0.114	−6.344^§^	0.338^*^	2.12
		Reduced	AR	70.638^§^	0.161^†^	1.321	−0.119	−1.442	− 0.340^†^	−5.002^§^	0.333^*^	2.14
		Constant	OLS	8.794^§^	0.301^§^	−1.792^§^	0.094^*^	0.426	0.220^§^	−1.370^§^	–	1.99
	Number of patients	Overall	OLS	1,408,058^§^	11,758^§^	− 47643^*^	1698	17,535	− 2504	−89,533^§^	–	1.95
		Reduced	OLS	1,129,486^§^	4909^§^	− 6604	532	7296	−7523^§^	−64,306^§^	–	2.07
		Constant	AR	278,489^§^	6820^§^	−39,690^§^	1207	8751	4989^§^	−26,186^§^	−0.168	1.79
Costs per patient (KRW)		Overall	OLS	43,492^§^	160^§^	− 2149^§^	− 163^§^	−3380^§^	107^*^	− 1063^§^	–	1.86
		Reduced	OLS	20,992^§^	−30^†^	1123^§^	−145^§^	−3670^§^	81^§^	− 550^§^	–	1.96
		Constant	AR	22,455^§^	183^§^	− 2800^§^	−27	233	43	− 499^†^	−0.485^†^	2.12
DDDs per patient		Overall	AR	94.160^§^	0.095^#^	0.317	−0.083	−1.564	− 0.010	−2.610^*^	0.224	2.12
		Reduced	AR	62.535^§^	−0.120^§^	1.330^†^	−0.090^*^	−1.416^†^	0.056	−1.384^§^	0.135	2.04
		Constant	OLS	31.594^§^	0.215^§^	−0.934	−0.0004	0.005	−0.068	−1.123^#^	–	2.08

*ß*_*0*_ = coefficients for the baseline levels of the outcome variables; *ß*_*1*_ = coefficients for the changes in the outcome variables before the OPIP, i.e. the baseline trends; *ß*_*2*_ = coefficients for the level changes in the outcome variables after the OPIP; *ß*_*3*_ = coefficients for the changes in the trend of the outcome variables after the OPIP; *ß*_*4*_ = coefficients for the level changes in the outcome variables after the SPS; *ß*_*5*_ = coefficients for the changes in the trend of the outcome variables after the SPS; and *ß*_*6*_ = coefficients for Feb variables

*OLS* ordinary least squares estimates, *AR* 1st order Autocorrelation Maximum likelihood estimates, *DW* Durbin-Watson *d* statistic, *KRW* Korean won (1000 KRW ≒ 1 US$ in January 2015), *DDD* defined daily dose

^#^ *p* < 0.1; ^*^ *p* < 0.05; ^†^ *p* < 0.01; ^§^ *p* < 0.001

In contrast, spending on antidiabetics_p-keep_ rose steeply by 42% from 12 to 17 billion KRW per month during the year after the SPS scheme, compared to a 19–20% increase during the baseline period (Table 2). A slope for the time series of antidiabetics_p-keep_ costs was increased from 0.28 to 0.49 billion KRW per month after the scheme was implemented (*β*_*5*_ = 0.211, *p* < 0.001, Table 3).

Collectively, the overall cost of antidiabetics was immediately reduced by 4.4 billion KRW in the month that the new pricing began, but showed a rising trend from 0.16 to 0.35 billion KRW per month afterwards (both *p* < 0.01, Table 3).

**Fig. 1:**
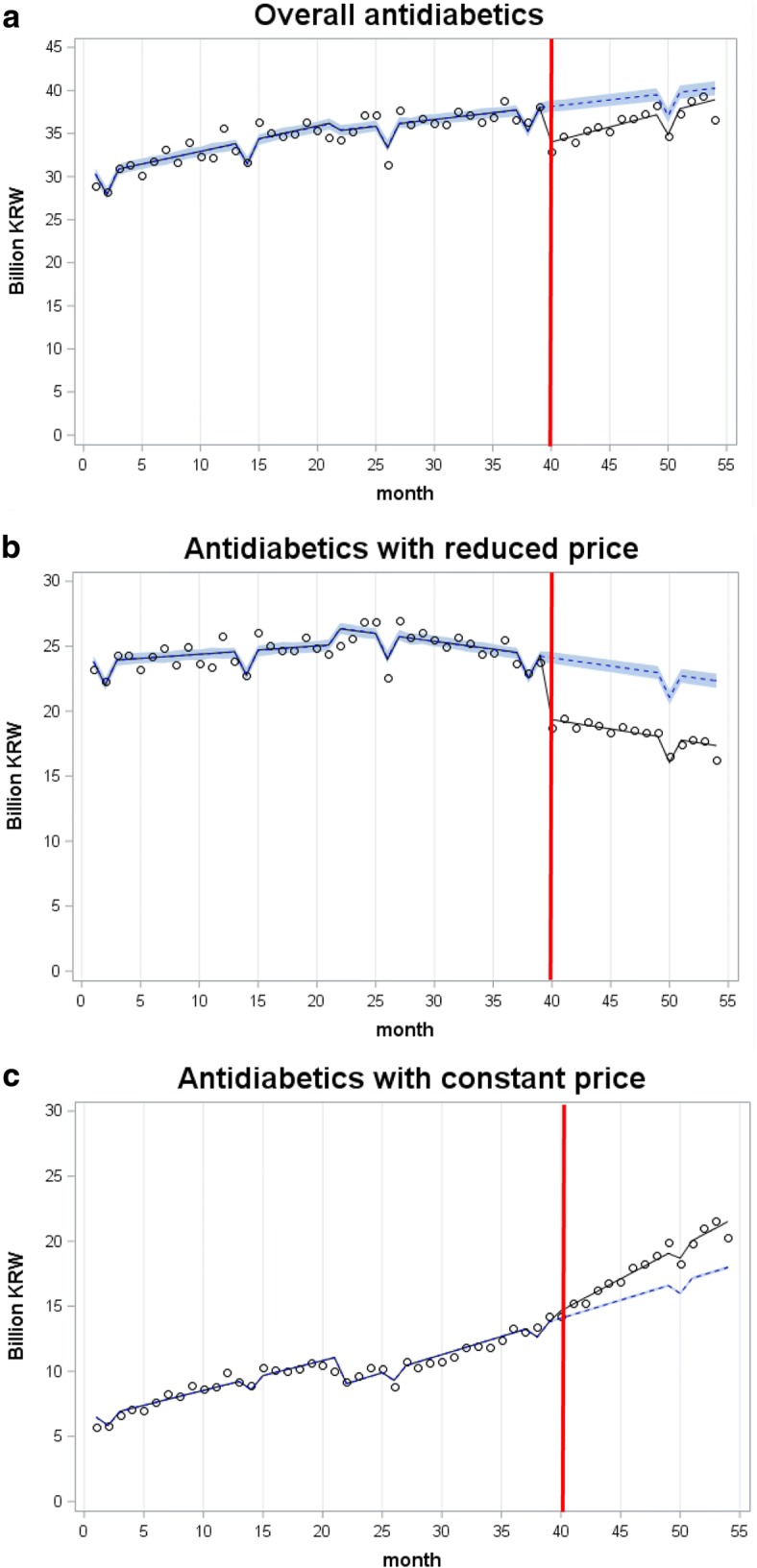
Observed and forecasted costs of antidiabetics. **a** Overall antidiabetics, **b** Antidiabetics with reduced price, **c** Antidiabetics with constant price. Gray band = 95% confidence interval; ° real values; −–– observed trend; −----- forecasted trend. KRW = Korean won (1000 KRW ≒ 1 US dollar)

Figure 1 shows the outcome measures for (a) cost of overall antidiabetics, (b) cost of antidiabetics with reduced prices (antidiabetics_p-cut_), and (c) cost of antidiabetics with constant prices (antidiabetics_p-keep_) along with each of the forecasted series with 95% confidence intervals. The segmented regression models suggest that the cost spent on overall antidiabetics and antidiabetics_p-cut_ would drop by 6 and 23%, respectively, in a year after the new pricing, compared to if the policy were not in existence. At the end point of the data period (the 15th month after the introduction of the policy), the rate of the decline was larger; 9% for overall antidiabetics (monthly average = 7%) and 27% for antidiabetics_p-cut_ (monthly average = 21%). In contrast, cost of antidiabetics_p-keep_ would rise by 16% in a year after the new pricing, compared to if the policy were not in existence. The rate of growth faded to 13% at the 15th month after the new pricing (monthly average = 13%).

### Effects on the utilization of antidiabetics

Overall antidiabetic use was not significantly affected by the price cut, but changes seen in antidiabetics_p-cut_ and antidiabetics_p-keep_ were the opposite. After the scheme was introduced, the increasing trends in the time series of antidiabetics_p-cut_ was reversed from 0.042 to − 0.298 million DDDs per month (*β*_*5*_ = − 0.340, *p* < 0.01; Table 3), and the increasing trends of the time series of antidiabetics_p-keep_ increased from 0.395 to 0.615 million DDDs per month (*β*_*5*_ = 0.220, *p* < 0.001; Table 3). The slope of the time series of the number of patients prescribed antidiabetics_p-cut_ was overturned from 5441 to − 2082 persons per month after the introduction of the scheme (*β*_*5*_ = − 7523, *p* < 0.001; Table 3). The slope of the number of patients in antidiabetics_p-keep_ increased from 8027 to 13,016 persons per month (*β*_*5*_ = 4989, *p* < 0.001; Table 3).

The segmented regression model suggests that the DDD utilization of overall antidiabetics and antidiabetics_p-cut_ would drop by 3 and 8% respectively in a year after the new pricing, compared to if the policy were not in existence. At the end point of the data period (the 15th month after the introduction of the policy), the rate of decline was larger; 8% for overall antidiabetics and 14% for antidiabetics_p-cut_. The segmented regression model suggests that the utilization of antidiabetics_p-keep_ would rise by 12% in a year after the new pricing, compared to if the policy were not in existence (monthly average = 2 and 5% respectively). The growth faded to 8% at the 15th month after the new pricing (monthly average = 9%).

### Effects on the cost and use of individual patients

After the SPS scheme, cost per patient for overall antidiabetics and antidiabetics_p-cut_ showed a significant and immediate reduction by 3380 and 3670 KRW, respectively (both *p* < 0.001). Cost per patient for overall antidiabetics switched to an increasing trend from − 2.1 to 104.8 KRW per month. The decreasing rate of cost per patient for antidiabetics_p-cut_ slowed down from − 174.8 to − 94.1 KRW per month. The changes per patient cost of antidiabetics_p-keep_ were found to be insignificant both in the level and the slope (*p* > 0.1; Table 3).

On a per patient basis, a 1.4 DDD drop was seen in antidiabetics_p-cut_ in the month when the new pricing was introduced (*p* < 0.05; Table 3). Besides this, no major changes were observed in the slope for the individual use of antidiabetics_p-cut_, or in the level and slope of overall antidiabetics or antidiabetics_p-keep_ (*p* > 0.1; Table 3).

### Incidents of medical and surgical procedures relating to diabetic complications

Table 4 displays the changes in monthly incidents of diabetes-induced medical and surgical procedures during the study period. The annual growth rate after the new pricing were 3–7% lower than those in the baseline period in all procedures that were examined.

**Table 4:** Monthly incidents of medical and surgical procedures relating to diabetic complications in the study cohort

	2009 April 2009 to March 2010	2010 April 2010 to March 2011		2011 April 2011 to March 2012		2012 April 2012 to March 2013	
	Episodes per 1000 patients	Episodes per 1000 patients	Annual growth (%)	Episodes per 1000 patients	Annual growth (%)	Episodes per 1000 patients	Annual growth (%)
diabetic retinopathy, monthly	0.27	0.29	+ 0.02 (7%)	0.30	+ 0.01 (3%)	0.30	0.0 (0%)
diabetic cataract, monthly	1.98	2.08	+ 0.1 (5%)	2.19	+ 0.11 (5%)	2.23	+ 0.04 (2%)
diabetic nephropathy, monthly	0.47	0.67	+ 0.2 (43%)	0.74	+ 0.07 (10%)	0.75	+ 0.01 (1%)
cardiovascular complications of diabetics, monthly	2.49	2.84	+ 0.35 (14%)	2.90	+ 0.06 (2%)	2.76	−0.14 (−5%)
diabetic foot lesions, monthly	0.40	0.53	+ 0.13 (33%)	0.62	+ 0.09 (17%)	0.68	+ 0.06 (10%)

## Discussion

The Korean government introduced the SPS, a direct price cut schedule in pharmaceutical pricing to contain pharmaceutical expenditure without causing any major negative changes in public health. Through analyzing the claims data, we found that a direct price cut contained costs during the study period by bringing an immediate cost reduction in the targeted pharmaceuticals. Additionally, we discovered that incidents of medical and surgical procedures relating to diabetic complications were unaffected or marginally reduced, suggesting the absence of any major effects on individual clinical outcomes during the study period. As individual utilization was only affected momentarily when the price of pharmaceuticals was reduced by the pricing policy, we cautiously expect few consequences in the long run, beyond the study period.

The savings was, however, expected to be offset by a prescription shift from reduced price pharmaceuticals (targeted) to constant price pharmaceuticals (non-targeted) in the long run. This was because antidiabetics with constant prices mostly included on-patent products with higher prices than the targeted antidiabetics, or new pharmaceutical entities uninfluenced by the new pricing policy. Similar phenomena had been observed in the case of antihyperlipidemic agents in Korea [35]. Extensive price cuts between 2008 and 2010 did not effectively contain the growth of pharmaceutical expenditures due to several factors, including the increased use of expensive drugs. Antihyperlipidemic agents without the price cuts showed increased expenditure and volume trends, which was also observed in antidiabetic agents in our analysis. Han et al. [36] also found that the price cut in antibiotics reduced pharmaceutical expenditures immediately, but the effect faded over the long run. Another study found that the price cut policy decreased expenditures of antihypertensive drugs, though the effect faded out over time [37]. The authors suggested that this might be because clinicians switched to pharmaceuticals with a constant price, which led to an unintended impact of increased drug utilization. The phenomenon of prescription shift from targeted to non-targeted products was in line with the results reported by Hsu et al. [38]. A shift of expenditure and utilization from “targeted” to “non-targeted” oral antidiabetics was seen after a reduction in drug reimbursement in Taiwan.

Rationally, few economic motivations exist for prescribers or dispensers to move from pharmaceuticals with reduced prices to those with constant prices in Korea. This is because the Korean government has not allowed any mark-up profits for healthcare providers in prescription pharmaceuticals since 1999, and healthcare providers have been rewarded only through service fees [27]. Pharmacies are separate facilities from clinical offices and physicians have no financial interests in pharmacies or pharmaceutical affairs. Physicians write a prescription, then patients are free to take the prescription to any pharmacy. Thus, in theory, pharmaceutical companies hardly influence health providers’ choices.

Notwithstanding, our study uncovered prescription shifts, which suggests that pharmaceutical companies reacted to the SPS with economic motives for profits. Pharmaceutical companies might have influenced health providers’ choices by replacing their reduced price products with other products outside of the range of the new pricing regulation. Of the 97 antidiabetic ingredients included in our analysis, eleven ingredients were introduced into the market just after the policy was introduced, and those eleven rapidly grew in cost by 20% per month between August 2012 and June 2013 based on our data. Pharmaceutical companies might have intensified marketing activities, which may have possibly affected healthcare providers’ prescription behavior towards constant price products. Meanwhile, the price difference between the reduced and the constant price products may not have been large enough for patients to stay with products with reduced prices when a healthcare provider suggested switching to a new one with a constant price.

Prescription shifts after the implementation of the SPS imply that the price cut for pharmaceuticals alone is not effective in controlling pharmaceutical expenditures in the long-run. Controlling pharmaceutical expenditures without sacrificing quality of care and adverse health outcomes requires other options such as additional cost control mechanisms on the demand side (i.e., tiered-benefit design, drug budget control [15], or more innovative programs such as value-based pricing).

To the best of our knowledge, this is the first study that measured the effects of the SPS on the cost and utilization of antidiabetic drugs and diabetic-induced medical and surgical procedure incidents through a rigorous quasi-experimental design. Unlike a previous study that examined the costs and utilization of antihypertensives with sample data that accounted for 1% of the Korean population [37], this study used the entire claims database. The Korean Diabetes Association [39] reported that the prevalence of diabetes was 10.1% in 2010, and about 3.2 million Koreans age 30 and above have diabetes. Another 3 million Koreans are at a prediabetic stage. Our cohort included about 4 million patients, which enclosed a comprehensive number of patients that was taking antidiabetics during the study period. In addition, different from Han et al. [36] that examined the impacts of the SPS on antibiotics during the nine months after policy implementation, we included data with a range of over a year, allowing us to control for seasonality factors, if any. Because pharmaceutical utilization can be seasonal, it is important to include data covering the whole year and test seasonality for the internal validity of the study [31].

However, there are some limitations to our study. We included claims from five major types of medical institutions (clinics, nursing homes, teaching hospitals, general hospitals, and other hospitals) and excluded those from dental hospitals/clinics, public healthcare centers, and herbal hospitals/clinics. Dental and herbal medical institutions were excluded because they were irrelevant in caring for diabetic patients. Public healthcare centers were excluded because they accounted for only a small portion of medical expenses, and healthcare providers were reimbursed in a different way from the institutions we examined. The five major types of medical institutions spent 81% of medical expenses for outpatient care in 2013 [28]. Thus, we expect that the excluded data may have limited the accuracy of our analysis but with only minor influence. We employed surrogate endpoints such as incidents of medical and surgical procedures to measure diabetic complications rather than examined clinical endpoints. This is an inherent limitation of the administrative claims data although we tried to include an exhaustive list of procedures related with diabetic complications. Patient level data investigation with clinical information will be necessary before concluding the effects of the SPS on public health.

## Conclusions

A direct price cut policy, the Single Price System (SPS), contained costs in the Korean pharmaceutical market for antidiabetics for a short period by bringing an immediate cost reduction in targeted pharmaceuticals. The saving was expected to be compensated by a prescription shift from reduced price pharmaceuticals to constant price pharmaceuticals in the long run. Trends of individual cost or utilization of antidiabetics were not significantly affected by the new policy. The effect of SPS was not evident to change rates of incidents of medical and surgical procedures. However, further research using clinical information is needed to conclude the clinical effect of SPS in patients with diabetes.

## Abbreviations

antidiabetics_p-cut_: Antidiabetics with reduced price
antidiabetics_p-keep_: Antidiabetics with constant price
AR: 1st order Autocorrelation Maximum likelihood estimate
ATC: WHO Anatomic Therapeutic Chemical group
DDD: Defined daily dose
DW: Durbin-Watson *d* statistic
HIRA: Health Insurance Review & Assessment Service
ICD-10: 10th version of the International Classification of Diseases
KRW: Korean Won
MedAid: Medical Aid
NHI: National Health Insurance
OLS: Ordinary least squares estimate
OPIP: Outpatient Prescription Incentive Program
SD: Standard deviation
SPS: Single Price System
